# A Community-Based Sensory Training Program Leads to Improved Experience at a Local Zoo for Children with Sensory Challenges

**DOI:** 10.3389/fped.2017.00193

**Published:** 2017-09-15

**Authors:** Michele Kong, Mallory Pritchard, Lara Dean, Michele Talley, Roger Torbert, Julian Maha

**Affiliations:** ^1^University of Alabama at Birmingham, Birmingham, AL, United States; ^2^Vestavia Hills, KultureCity, Birmingham, AL, United States; ^3^Birmingham Zoo, Birmingham, AL, United States

**Keywords:** sensory sensitivities, sensory dysregulation, sensory training, zoo, community, autism, special needs, inclusion

## Abstract

Sensory processing difficulties are common among many special needs children, especially those with autism spectrum disorder (ASD). The sensory sensitivities often result in interference of daily functioning and can lead to social isolation for both the individual and family unit. A quality improvement (QI) project was undertaken within a local zoo to systematically implement a sensory training program targeted at helping special needs individuals with sensory challenges, including those with ASD, Down’s syndrome, attention-deficit/hyperactivity disorder, and speech delay. We piloted the program over a 2-year period. The program consisted of staff training, provision of sensory bags and specific social stories, as well as creation of quiet zones. Two hundred family units were surveyed before and after implementation of the sensory training program. In this pilot QI study, families reported increased visitation to the zoo, improved interactions with staff members, and the overall quality of their experience. In conclusion, we are able to demonstrate that a sensory training program within the community zoo is feasible, impactful, and has the potential to decrease social isolation for special needs individuals and their families.

## Introduction

Sensory processing difficulties are common among many special needs children (1–3). It is becoming more widely recognized that many of the challenges experienced by these children in public places are a result of their inability to process the incoming sensory input. While all younger children can seem particular about their likes and dislikes, children with sensory sensitivities can be so severely affected by their sensory preferences that it interferes with their everyday functioning (4). For instance, children with hyper-responsiveness to sound may have an atypical response such as covering of their ears in response to everyday noise, while those with an atypical response to light may squint or cover their eyes when in a bright room (5). For those with sensory gating challenges, the brain may not able to filter out redundant responses to irrelevant environmental stimuli, potentially leading to an overwhelmed sensory system (6).

Additionally, children with more significant sensory abnormalities have also been shown to have more behavioral problems (7). In a recent study by Mazurek et al., the investigators reported increased behavior issues and family impairment in children with sensory hyper-responsiveness (8). Parents of children with higher over-sensory sensitivity were also found to have increased parental strain (9). Ultimately, visits to public facilities such as zoos, restaurants, concert halls, and participation in public events become extremely difficult, contributing to social isolation for both the individuals and their family unit (10).

A quality improvement (QI) project was undertaken at the Birmingham National Zoo in Birmingham, AL, USA^1^ to improve the current practices when encountering a family or visitor with a sensory need. We hypothesized that the sensory training program will lead to increased participation, engagement, and quality of experience for special needs individuals and their families. The program was implemented over a 2-year period, and we were able to successfully show that participation in the sensory training and consistent execution resulted in a positive outcome and impact within the community.

## Materials and Methods

### Subject Selection and Survey

Informational flyers were distributed to local schools and therapy programs that served special needs individuals within the Birmingham area. The first two hundred families who responded to the call for participation were contacted, and given a survey (*via* www.surveymonkey.com). All the families had at least one child within the household with either autism spectrum disorder (ASD), Down’s syndrome, cerebral palsy, or other special needs diagnosis. Before implementation of the sensory program, survey questions sent to the families included the frequency of visits to the zoo, barriers encountered at the zoo, quality of staff interaction, and services provided (Appendix A in Supplementary Material). At the end of the zoo sensory training program, a post survey was distributed to all participating families. Post training survey questions included frequency of visit to the zoo, quality of staff interaction, frequency of usage for sensory bags, and quiet zones (Appendix B in Supplementary Material).

### Sensory Training Program

A sensory training program was developed and provided to all zoo staff members (any staff and volunteer who would come into contact with a zoo guest). The entire program was implemented over a 2-year period, from 2015 to 2017. The training was provided by a team consisting of a physician, an occupational therapist, a speech therapist, and a behavioral therapist covering topics such as presentations of sensory overload, best methods to engage and communicate, and techniques to handle issues resulting from a sensory overload. Over the course of the program, a total of four main training sessions were given to the entire zoo staff. In addition, throughout the year, smaller informal sessions were held, especially during the onboarding process of new hire and volunteers. In addition to the staff training, we also created a sensory bag containing a noise canceling headphone, fidget tools, visual cue card, and a weighted lap pad. These bags were made available to the special needs individuals at no cost during the zoo visit upon request. Families also had access to a social story (a short story describing the zoo visit, written from a child’s perspective) that was created specifically for the Birmingham Zoo. Finally, modifications were made to specific parts of the zoo (for instance, designation of quiet zones) to help provide an additional layer of service for those with sensory sensitivities.

### Qualitative Analysis Methods

In order to further determine the potential significance and utility of the sensory training program, we also sought to determine detailed qualitative data from the families regarding the impact of the sensory training program on their family. Families were given the opportunity to provide feedback and leave comments regarding their experience before and after the implementation of the sensory training program. The comments from one pre-training (Appendix A, Question 6 in Supplementary Material) and one post-training question (Appendix B, Question 9 in Supplementary Material) were compiled for analysis. Before the launch of the program, the parents were asked if they had implemented any strategies to decrease anxiety before the zoo visit. Those parents who said yes, left comments, and these comments were analyzed. Likewise, after the launch of the program, the parents were asked to provide any comments they wished.

NVivo Pro version 11 software was used to analyze the data. First, all of the compiled comments were analyzed. A word frequency query was run. The most commonly used words by the families in response to the questions appeared in a report within NVivo. These words were sorted for appropriateness to the question asked. Next, these words were sorted into synonym groups (e.g., words such as “help, helpful, helping, and service” counted toward the frequency of the word “help”). The size of the word located within the word cloud was determined by the frequency that the word(s) were used. The words that were used the most appeared larger than those words that were cited less often. Only common words and words that did not fit the context were excluded.

The frequency of the words that were used to answer Question 6 (Appendix A in Supplementary Material) before the launch of the program was reported (Appendix C, Table S1 in Supplementary Material) and analyzed. As a result, the first word cloud was produced (Image S1 in Supplementary Material). The frequency of the words that was used to answer Question 9 (Appendix B in Supplementary Material) after the launch of the program were reported (Appendix D, Table S2 in Supplementary Material) and analyzed. As a result, the second word cloud was produced (Image S2 in Supplementary Material).

The compiled comments for both questions were also coded for common themes. The common themes were grouped according to whether they were made before or after the launch of the sensory training program. Multiple readings of the raw data yielded interpretations. Categories related to these interpretations were identified and key themes were captured as a model or framework.

### Ethics

This project was undertaken as a QI activity to systematically teach and implement our current existing knowledge of sensory overload and regulation in special needs individuals. There is on going direct benefit to the community as a whole, and no increased risk for participation. Guidelines for reporting QI initiatives published by the SQUIRE Development Group were consulted for this manuscript (11).

### Consent

This study was carried out with voluntary and electronic informed consent from all study participants. Survey respondent anonymity was ensured by not collecting any personally identifiable information such as name, home, email, or IP address. All data were stored in a password-protected server. No minors were involved in this study.

## Results

### Demographics

Two hundred special needs families or households were included in this survey. In order to participate in the study, families had to have at least one child with a special needs diagnosis. Table 1 outlines the number of children per household with sensory needs, age, gender, and medical diagnosis of the special needs children and individuals surveyed. In total, we had 230 individuals within this study (as some families had more than one special needs child), with 289 data point for diagnosis (many individuals had more than one primary diagnosis).

**Table 1:** Demographic of the 200 special needs families/households.

Number of children per household	*n*	%	Total *n*
1	158	79	
2	31	15.5	
3	11	5.5	200
**Age**			
9–12 years old	70	30.4	
13–16 years old	23	10	
17–21 years old	10	4.3	
22 years and older	3	1.2	230
**Gender**			
Female	58	26.7	
Male	159	73.3	217
**Medical diagnosis**			
Autism spectrum disorder	161	55.7	
Attention-deficit/hyperactivity disorder	61	21.1	
Cerebral palsy	6	2.1	
Downs syndrome	15	5.2	
Other^a^	44	15.2	
Not disclosed	2	0.7	289

*^a^Speech delay, epilepsy, sensory processing disorder, apraxia, developmental delay, chromosomal abnormality, anxiety, post traumatic stress disorder, auditory processing disorder, tuberous sclerosis, fragile X, spina bifida, oppositional defiant disorder, obsessive–compulsive disorder, deafness, and tourettet’s syndrome*.

### Assessment of Need

In the pre-training survey, 83% of the families reported that their child frequently had issues with sensory overload when visiting the zoo. Overwhelming noise levels, long wait lines, and poor interaction with staff members were among the reported barriers to a successful visit. Approximately half of the families implemented strategies to decrease the level of anticipated anxiety for their children prior to the zoo visit. Strategies implemented include pre-planning and talking about the trip, going during anticipated low attendance times, following a schedule, creating breaks, avoiding certain exhibits and bringing their headphones, fidget toys, and snacks (Image S1 in Supplementary Material).

### Frequency of Zoo Visitation

**Figure 1:**
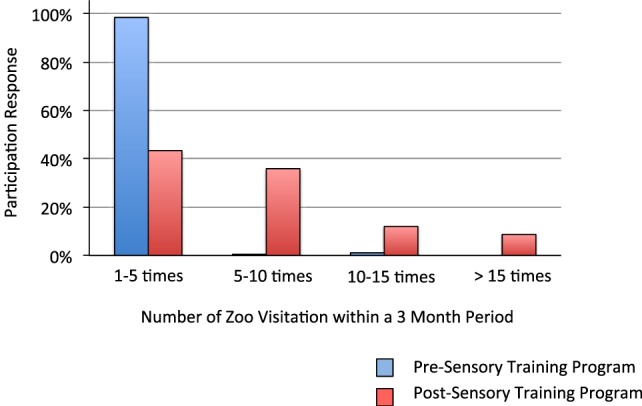
Increase in zoo attendance after implementation of the sensory training program.

Prior to implementation of the sensory training program, 98.4% of the families surveyed reported that they visited the zoo 1–5 times within a 3-month period. 0.5 and 1.1% of the families reported visiting the zoo 5–10 times, and 10–15 times within that time frame, respectively. In contrast, the number of zoo visitations increased significantly post-implementation of training with 43.4% reporting that they visited the zoo less than five times within a similar time frame. 35.9, 12, and 8.7% reported visiting the zoo 5–10 times, 10–15 times, and greater than 15 times respectively (Figure 1).

### Staff Interaction with Families

Prior to the sensory training, 42% of the families reported no interaction with the zoo staff. 18% reported poor to fair interactions, 30% reported a good interaction with only 10% reporting an excellent level of interaction. Post sensory training, the level of satisfaction with staff members increased dramatically. Only 15% reported no interactions and 21% reported poor to fair interactions. 41% of the families reported a good interaction, while 23% reported an excellent level of staff interactions.

Sensory bags: 42.4% of special needs individuals utilized the sensory bag during their zoo visits. 65.8 and 56.1% reported that fidget tools and noise canceling headphones, respectively, were beneficial items within the bag. Only 8% of families reported use weighted lap pads during shows and train rides and found them to be of utility.

### Quiet Areas

56% of the families reported use of the designated quiet areas within the zoo to prevent sensory overload or when the individual was having a sensory meltdown. Quiet areas were locations within the zoo that typically had less activity with spaces to sit for those in need to regroup and calm.

### Was Sensory Needs Met?

More than 70% of the special needs individuals and families surveyed reported that their sensory needs were met after implementation of the sensory training. 27.8% reported that their needs were met, but suggested additional modifications (such as provision of gluten-free menus in the café, and use of paper towels vs. electronic hand dryer in the bathrooms). Only 2.2% of those surveyed said that their sensory needs were not sufficiently met.

### Qualitative Analysis Results

For the first qualitative analysis, a word cloud was formed for the strategies the families used before the launch of the visit (Image S1 in Supplementary Material). A second word cloud was formed using the words most frequently used by the families after the launch of the training (Image S2 in Supplementary Material).

For the second qualitative analysis, themes before the launch of the program and after the launch of the program revealed separate themes. Before the sensory training program, several independent themes emerged as strategies that the parents utilized before visiting the zoo. These themes included: (a) avoidance of the Birmingham Zoo during specific visitation times; (b) avoiding certain areas of the zoo; (c) following a schedule and having a plan; (d) taking frequent breaks; (e) using social stories and talking to the child about the experience; (f) using headphones, strollers, chewy, and fidget toys. Post sensory training, the themes that emerged included: (a) families being able to participate as a unit; (b) the sense of community; (c) increased awareness for sensory issues; (d) increased acceptance for their children; (e) the help given to each child.

## Discussion

Over the last few years, there has been an increase in our awareness of the challenges faced by individuals with sensory sensitivities. Although sensory processing disorders has been reported to affect up to 16% of children (1), the implications and impact of sensory dysregulation is much more significant. This is because sensory dysregulation is also prevalent in children with other medical diagnosis, for instance those with ASD, fragile X syndrome, seizure disorders, obsessive–compulsive disorder, attention-deficit/hyperactivity disorder, or those who are premature (3, 12–17). In ASD alone, recent estimates suggest a prevalence of up to 96% of autistic children who have abnormal sensory responses. Many can have impairments in intellectual and social development from disrupted processing and integration of information from the different sensory modalities. For instance, a child may be overtly sensitive to everyday stimuli (e.g., the sound of a flushing toilet or hand dryer in the bathroom, brightness in a room), or have a negative reaction to a sensory stimulus not typically considered aversive (e.g., tag on clothes or a touch) (18). Senses that are either over or under-reactive to stimulation may also be the basis of certain behaviors such as jumping, rocking, spinning, or hand flapping (19). In fact, great progress has been made in our understanding of the sensory differences in these children, including correlations between clinical assessments of sensory dysfunction with the underlying neural mechanistic causes (20–22). Often, for all these individuals, having an inappropriate response to sensory inputs is manifested as emotional and behavioral disruptions, leading to interruptions in their daily functioning and potentially social isolation.

We were able to show that a sensory training program is feasible and have the potential for significant impact within the special needs community. Up to 83% of the families surveyed reported having at least one individual within their family unit with a sensory need. These individuals had a wide range of medical diagnosis, ranging from ASD, Down’s syndrome, seizure disorders to post-traumatic stress disorder. More than 47% of families reported that they were utilizing different methods to decrease the level of anxiety and to meet the sensory needs of their children even prior to the implementation of training. However, despite the various methods used by the families, 98% of families reported that they only visited the zoo between 1 and 5 times annually due to the sensory challenges faced by their child. Furthermore, pre-training, up to 60% of families reported none to fair levels of interactions with zoo members.

Our finding of low attendance and avoidance of certain public places by families of special needs children, particularly with ASD and sensory sensitivities is consistent with what is currently found in the literature. It is widely recognized that families of children with ASD and other developmental disabilities experience a higher level of strain and stress compared to families with typically developing children (23, 24). Recently, Bagby et al. reported that parents of children with sensory challenges tend to limit or avoid certain places because of their child’s sensitivities (25). In another study, sensory behaviors were found to impact daily routines, and were identified as an important factor that limited participation in leisure activities (10). Due to the abnormal sensory integration and the implications on the individual and family unit, social isolation and social deficits in this particular group of individual is commonplace (26). The limited participation and avoidance of particular activities is important due to its long-term implications on the child and family (23–27). In this pilot study, we were able to show that implementation of a sensory training program within a community-based facility lead to increased attendance and participation from families.

By making the Birmingham Zoo sensory inclusive, families are now able to enjoy the zoo amenities at any time without being limited to special zoo events that are targeted toward the special needs community. The biggest barrier toward inclusion is removed simply by educating the general public, specifically in this pilot QI study, the zoo staff on what it means to have sensory processing challenges, ways to recognize sensory overload, and different methods to help mitigate some of these issues. By simply increasing the understanding of the underlying and significance of sensory sensitivities, the quality of service is improved. It is remarkable to note that with a systematic approach, and with training of staff members, more than 97% of the families surveyed reported that the sensory needs of their children were met.

Before implementation of the training program, many of the families were utilizing different strategies including bringing their own headphones, fidget toys, and using social story. In trying to minimize potential for sensory overload and meltdown, families avoided visitation during busy hours of the day, or only visited during special events. Despite individual efforts by these families, levels of participations before implementation of the sensory training program were low. By utilizing a systematic approach, and importantly, the staff training, we found that many of the barriers previously encountered were mitigated, and ensured a successful visit, and improved experience at the zoo. In fact, post training, 64% of families reported good to excellent levels of interactions with the staff. The level of interactions were reported to be improved because the staff members were more equipped and were able to utilized best methods to communicate and calm. Furthermore, having a quiet space was also felt to be beneficial as it allowed for the individuals to decompress when having an overwhelming sensory experience. Having the ability to regulate and decompress without having to leave the zoo allowed them to return to their zoo visit once the episode is resolved.

The main challenge in implementing this sensory training program was ensuring that all the staff members were adequately trained, especially with the turnover related to seasonal volunteers. We met this challenge by having smaller informal sessions used especially during the onboarding process. We also created an online module that the staff can access for further training.

This study has several limitations. The families were selected to participate in this Zoo QI initiative based on their self-identification of having a child with special needs. We did not confirm the child’s medical diagnosis or require medical proof of the diagnosis. The sensory overload or dysregulation experienced by the child is based on subjective reports given by the family. Since this was a QI project that included training of all the zoo staff, and any modifications made were accessible to all special needs individual visiting the zoo, we did not have a control group in this study. It is possible that the families who participated in the survey did other interventions during the study period, or that the child became more tolerant of public spaces with time. However, it is important to note that in addition to increase attendance, families also reported improved satisfaction with staff interactions, and more than 70% felt that the sensory need of the child was met during the zoo visit.

Most importantly, after the implementation of the sensory training program, the biggest reported trend that emerged was the feeling of acceptance and inclusion by the families, whereby the zoo was felt to be a safe place, and that their child was not judged when having a sensory meltdown. Quotes such as these were given by families “*I loved the ability to come to the zoo*…*. It was the first time in 20 years that my family and I felt comfortable at an event where we knew if my son was to have a meltdown or make strange sounds, we would not be stared at or the fact that my son although a big guy but still sees the world through the eyes of a 5-year old*.”

## Conclusion

We showed that a community-based sensory training program was feasible and impactful. This training program is also sustainable, as the Birmingham Zoo has remained sensory inclusive to date.^2^ The effects of the training program most likely also benefited the other families not in the study as reflected by the overall increase in zoo attendance. We were able to demonstrate that training of staff members, provision of sensory bags, and creation of quiet zones met the challenges of special needs children with sensory sensitivities. Our goal is to extend training program to other public facilities in order to validate our findings with a larger scale population.

## Ethics Statement

*Ethics*: This project was undertaken as a QI activity to systematically teach and implement our current existing knowledge of sensory overload and regulation in special needs individuals. There is on going direct benefit to the community as a whole, and no increased risk for participation. Guidelines for reporting QI initiatives published by the SQUIRE Development Group were consulted for this manuscript (11). *Consent*: This study was carried out with voluntary and electronic informed consent from all study participants. Survey respondent anonymity was ensured by not collecting any personally identifiable information such as name, home, email, or IP address. All data were stored in a password-protected server. No minors were involved in this study.

## Author Contributions

MK and JM contributed to the conception, data analysis, writing, and editing of this manuscript. MT contributed to the data analysis and editing of the manuscript. MP, LD, and RT contributed to the conception and data analysis. All authors approved of the final submitted version of this manuscript.

## Conflict of Interest Statement

The authors declare that the research was conducted in the absence of any commercial or financial relationships that could be construed as a potential conflict of interest.

## Footnotes

^1^Available from: https://www.birminghamzoo.com

^2^Available from: https://www.birminghamzoo.com/visitor-info/accessibility (accessed September 1, 2017).
