# Supplementary material for: A Community-Based Sensory Training Program Leads to Improved Experience at a Local Zoo for Children with Sensory Challenges
Source: Front Pediatr. 2017 Sep 15;5:193. doi: 10.3389/fped.2017.00193 (PMC5605637; doi:10.3389/fped.2017.00193)

**APPENDIX**

**A. Survey questions distributed to special needs families prior to the initiation of the Birmingham Zoo sensory training program**

1. How many children do you have in your household with sensory needs?

2. What is the age of your child with sensory needs?

3. Is your child male or female?

4. Does your child have one of the following diagnoses? Autism Spectrum Disorder, ADHD, Cerebral Palsy, Down’s Syndrome, Other (open comment)

5. How many times have you visited the Birmingham Zoo in the last three months?

6. Have you implemented strategies to decrease anxiety associated with your child’s visit to the zoo? If answered yes, please elaborate below.

7. Does your child have difficulty waiting in lines while at the zoo? (Waiting to enter zoo, get into the Safari cafe, getting on rides, etc.)

8. Does your child ever experience sensory overload or difficulty with regulation while at the zoo?

9. How would you rank the zoo staff's interaction with your child? No interaction at all; Poor; Fair; Good; Excellent.

**B. Survey questions distributed to special needs families at the end of the Birmingham Zoo sensory training program**

**1.** How many times have you visited the Birmingham Zoo in the last 3 months?

2. How would you rank the zoo staff's interaction with your child and family? No interaction at all; Poor; Fair; Good; Excellent.

3. Have you checked out a sensory bag for your child to utilize while at the zoo?

4. Which items in the sensory bag have you found to be beneficial for your child's zoo experience?

5. Have you checked out a weighted lap pad while viewing the Wildlife Show, Sea Lion Splash Show or riding the train?

6. Have you utilized the quiet areas for your child with sensory processing needs?

7. How would you rank the zoo staff's ability to answer questions about sensory modifications being made at the zoo? Poor; Fair; Good; Excellent.

8. Do you feel your child's sensory processing needs are being met during your trips to the zoo?

9. Are there any additional changes, modifications, services that we could offer to improve your families' zoo experience? Additional comments?

**C. Table 1: Word frequency that generated Image 1**

| **Word** | **Length** | **Count** | **Weighted Percentage (%)** | **Similar Words** |
| --- | --- | --- | --- | --- |
| times | 5 | 16 | 4.88 | multiple, time, times, timing |
| talking | 7 | 12 | 3.66 | talk, talking, verbal, verbally |
| visit | 5 | 12 | 3.66 | visit, visiting, visits |
| avoid | 5 | 11 | 3.35 | avoid, avoiding |
| breaks | 6 | 9 | 2.74 | breaks, wearing |
| schedule | 8 | 9 | 2.74 | schedule, schedules, scheduling |
| animals | 7 | 8 | 2.44 | animal, animals, physical |
| areas | 5 | 8 | 2.44 | area, areas |
| plan | 4 | 8 | 2.44 | plan, planned, planning, plans |
| social | 6 | 8 | 2.44 | social |
| stories | 7 | 8 | 2.44 | stories, story |
| stroller | 8 | 8 | 2.44 | stroller |
| help | 4 | 7 | 2.13 | help, helps, portion, service |
| snacks | 6 | 7 | 2.13 | snack, snacks |
| pictures | 8 | 7 | 1.98 | picture, pictures, show, visual |
| allow | 5 | 6 | 1.83 | allow, allowed, allowing, leaving |
| calm | 4 | 6 | 1.83 | calm, calmly, quiet |
| headphones | 10 | 6 | 1.83 | headphones |
| early | 5 | 5 | 1.52 | early |
| map | 3 | 5 | 1.52 | map |
| days | 4 | 4 | 1.22 | days |
| explaining | 10 | 4 | 1.22 | explain, explaining |
| fidget | 6 | 4 | 1.22 | fidget, fidgets |
| food | 4 | 4 | 1.22 | food |
| hold | 4 | 4 | 1.22 | book, hold |
| noise | 5 | 4 | 1.22 | noise, noises |
| sensory | 7 | 4 | 1.22 | sensory |
| exhibits | 8 | 4 | 1.07 | exhibits, show |
| blanket | 7 | 3 | 0.91 | blanket |
| chewy | 5 | 3 | 0.91 | chewie, chewy |
| discussing | 10 | 3 | 0.91 | discuss, discussing |
| items | 5 | 3 | 0.91 | items |
| odors | 5 | 4 | 0.91 | odors, smells |
| riding | 6 | 3 | 0.91 | riding, sit, sitting |
| toy | 3 | 3 | 0.91 | toy |
| trip | 4 | 3 | 0.91 | trip |
| weighted | 8 | 3 | 0.91 | weighted |
| ipad | 4 | 3 | 0.91 | ipad |
| advance | 7 | 2 | 0.61 | advance |
| ahead | 5 | 2 | 0.61 | ahead |
| anxiety | 7 | 2 | 0.61 | anxiety |
| bottle | 6 | 2 | 0.61 | bottle |
| carousel | 8 | 2 | 0.61 | carousel |
| comfort | 7 | 2 | 0.61 | comfort, comfortable |
| distract | 8 | 2 | 0.61 | distract |
| drinks | 6 | 2 | 0.61 | drinks |
| hours | 5 | 2 | 0.61 | hours, minutes |
| ipod | 4 | 2 | 0.61 | ipod |
| pace | 4 | 2 | 0.61 | pace, stepped |
| plastic | 7 | 2 | 0.61 | plastic |
| restroom | 8 | 2 | 0.61 | restroom, restrooms |
| smells | 6 | 3 | 0.61 | smells |
| space | 5 | 2 | 0.61 | space, spaces |
| walking | 7 | 2 | 0.61 | walking |
| animatronic | 11 | 1 | 0.30 | animatronic |
| bag | 3 | 1 | 0.30 | bag |
| benches | 7 | 1 | 0.30 | benches |
| board | 5 | 1 | 0.30 | board |
| cancellation | 12 | 1 | 0.30 | cancellation |
| carrier | 7 | 1 | 0.30 | carrier |
| climbing | 8 | 1 | 0.30 | climbing |
| communicating | 13 | 1 | 0.30 | communicating |
| computer | 8 | 1 | 0.30 | computer |
| cup | 3 | 1 | 0.30 | cup |
| definite | 8 | 1 | 0.30 | definite |
| device | 6 | 1 | 0.30 | device |
| diet | 4 | 1 | 0.30 | diet |
| distress | 8 | 1 | 0.30 | distress |
| dog | 3 | 1 | 0.30 | dog |
| earlier | 7 | 1 | 0.30 | earlier |
| electronic | 10 | 1 | 0.30 | electronic |
| environment | 11 | 1 | 0.30 | environment |
| explanation | 11 | 1 | 0.30 | explanation |
| family | 6 | 1 | 0.30 | family |
| feed | 4 | 1 | 0.30 | feed |
| flashcards | 10 | 1 | 0.30 | flashcards |
| games | 5 | 1 | 0.30 | games |
| hydrated | 8 | 1 | 0.30 | hydrated |
| indoor | 6 | 1 | 0.30 | indoor |
| informed | 8 | 1 | 0.30 | informed |
| labeled | 7 | 1 | 0.30 | labeled |
| medication | 10 | 1 | 0.30 | medication |
| minimize | 8 | 1 | 0.30 | minimize |
| monitor | 7 | 1 | 0.30 | monitor |
| morning | 7 | 1 | 0.30 | morning |
| moving | 6 | 1 | 0.30 | moving |
| nothing | 7 | 1 | 0.30 | nothing |
| oils | 4 | 1 | 0.30 | oils |
| plugs | 5 | 1 | 0.30 | plugs |
| prep | 4 | 1 | 0.30 | prep |
| prescription | 12 | 1 | 0.30 | prescription |
| pressure | 8 | 1 | 0.30 | pressure |
| problems | 8 | 1 | 0.30 | problems |
| proximity | 9 | 1 | 0.30 | proximity |
| redirecting | 11 | 1 | 0.30 | redirecting |
| review | 6 | 1 | 0.30 | review |
| route | 5 | 1 | 0.30 | route |
| shorten | 7 | 1 | 0.30 | shorten |
| slow | 4 | 1 | 0.30 | slow |
| sunglasses | 10 | 1 | 0.30 | sunglasses |
| tablet | 6 | 1 | 0.30 | tablet |
| telling | 7 | 1 | 0.30 | telling |
| therapy | 7 | 1 | 0.30 | therapy |
| vest | 4 | 1 | 0.30 | vest |
| wagon | 5 | 1 | 0.30 | wagon |
| water | 5 | 1 | 0.30 | water |
| wheelchair | 10 | 1 | 0.30 | wheelchair |
| wristband | 9 | 1 | 0.30 | wristband |

**D. Table 2: Word frequency that generated Image 2**

| Word | Length | Count | Weighted Percentage (%) | Similar Words |
| --- | --- | --- | --- | --- |
| family | 6 | 11 | 8.21 | families, family |
| help | 4 | 8 | 5.97 | help, helpful, helping, service |
| sensory | 7 | 8 | 5.97 | sensory |
| issues | 6 | 7 | 5.22 | event, events, issues |
| community | 9 | 6 | 4.48 | communities, community |
| parents | 7 | 6 | 4.48 | parent, parents, raising |
| changed | 7 | 4 | 2.99 | changed, changes, changing, modifications |
| world | 5 | 4 | 2.99 | world |
| everything | 10 | 4 | 2.99 | everything |
| acceptance | 10 | 3 | 2.24 | acceptance |
| awareness | 9 | 3 | 2.24 | aware, awareness |
| made | 4 | 3 | 2.24 | made |
| noise | 5 | 3 | 2.24 | noise, noises |
| time | 4 | 3 | 2.24 | time, times |
| headphones | 10 | 3 | 1.87 | headphones, phone |
| offer | 5 | 3 | 1.87 | offer, provided |
| bag | 3 | 2 | 1.49 | bag, bags |
| connect | 7 | 2 | 1.49 | connect, continue |
| friendly | 8 | 2 | 1.49 | friendly |
| hope | 4 | 2 | 1.49 | hope |
| kids | 4 | 2 | 1.49 | kids |
| opportunity | 11 | 2 | 1.49 | opportunity |
| program | 7 | 2 | 1.49 | program |
| allowed | 7 | 2 | 1.12 | allowed, provided |
| calls | 5 | 2 | 1.12 | calls, phone |
| ability | 7 | 1 | 0.75 | ability |
| anywhere | 8 | 1 | 0.75 | anywhere |
| canceling | 9 | 1 | 0.75 | canceling |
| carrying | 8 | 1 | 0.75 | carrying |
| chat | 4 | 1 | 0.75 | chat |
| comfortable | 11 | 1 | 0.75 | comfortable |
| early | 5 | 1 | 0.75 | early |
| enabled | 7 | 1 | 0.75 | enabled |
| everyday | 8 | 1 | 0.75 | everyday |
| excited | 7 | 1 | 0.75 | excited |
| experience | 10 | 1 | 0.75 | experience |
| explain | 7 | 1 | 0.75 | explain |
| eyes | 4 | 1 | 0.75 | eyes |
| fact | 4 | 1 | 0.75 | fact |
| felt | 4 | 1 | 0.75 | felt |
| fun | 3 | 1 | 0.75 | fun |
| intervention | 12 | 1 | 0.75 | intervention |
| introduced | 10 | 1 | 0.75 | introduced |
| items | 5 | 1 | 0.75 | items |
| mentally | 8 | 1 | 0.75 | mentally |
| morning | 7 | 1 | 0.75 | morning |
| night | 5 | 1 | 0.75 | night |
| occasions | 9 | 1 | 0.75 | occasions |
| ride | 4 | 1 | 0.75 | ride |
| safari | 6 | 1 | 0.75 | safari |
| show | 4 | 1 | 0.75 | show |
| staff | 5 | 1 | 0.75 | staff |
| teaching | 8 | 1 | 0.75 | teaching |
| words | 5 | 1 | 0.75 | words |
| anything | 8 | 1 | 0.75 | anything |
| rode | 4 | 1 | 0.75 | rode |

**Image 1: Word cloud representing strategies utilized by families to assist with sensory challenges during their zoo visitation prior to implementation of the sensory training program**


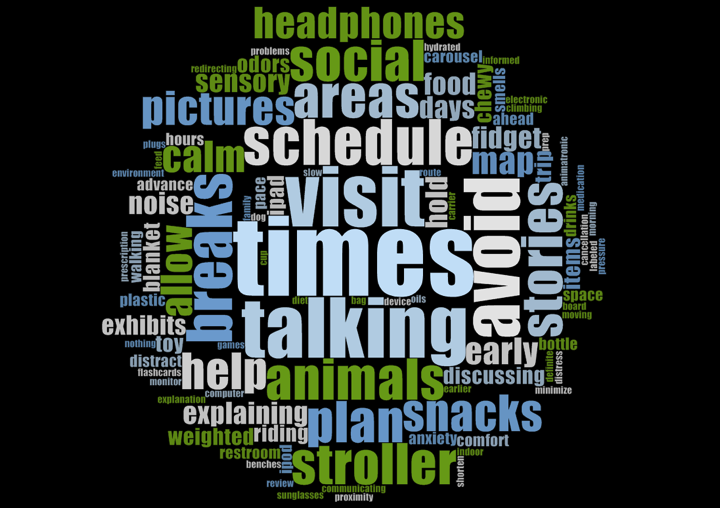


**Image 2: Word cloud representing themes that emerged from family response after the implementation of the sensory training program**


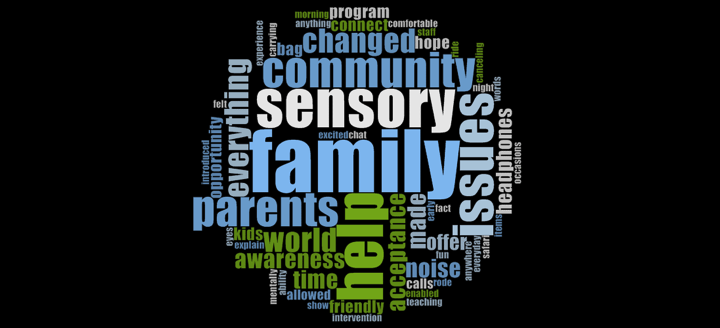

Supplement: Supplementary file 1 [file Data_Sheet_1.docx]
